# Integration of MsrB1 and MsrB2 in the Redox Network during the Development of Orthodox and Recalcitrant *Acer* Seeds

**DOI:** 10.3390/antiox9121250

**Published:** 2020-12-09

**Authors:** Ewelina Stolarska, Karolina Bilska, Natalia Wojciechowska, Agnieszka Bagniewska-Zadworna, Pascal Rey, Ewa M. Kalemba

**Affiliations:** 1 Institute of Dendrology, Polish Academy of Sciences, Parkowa 5, 62-035 Kórnik, Poland; ewelina.stolarska89@gmail.com (E.S.); mgr.karolina.bilska@gmail.com (K.B.); natalia.wojciechowska@amu.edu.pl (N.W.); 2 Department of General Botany, Institute of Experimental Biology, Faculty of Biology, Adam Mickiewicz University, Uniwersytetu Poznańskiego 6, 61-614 Poznań, Poland; agabag@amu.edu.pl; 3 Plant Protective Proteins (PPV) Team, Aix Marseille University (AMU), Commissariat à l’Energie Atomique et aux Energies Alternatives (CEA), Centre National de la Recherche Scientifique (CNRS), Biosciences and Biotechnology Institute of Aix-Marseille (BIAM), F-13108 Saint Paul-Lez-Durance, France; pascal.rey@cea.fr

**Keywords:** *Acer platanoides*, *Acer pseudoplatanus*, ascorbate, glutathione, seed maturation, methionine sulfoxide, methionine sulfoxide reductase, nicotinamide adenine dinucleotide phosphate

## Abstract

Two related tree species, Norway maple (*Acer platanoides* L.) and sycamore (*Acer pseudoplatanus* L.), produce desiccation-tolerant (orthodox) and desiccation-sensitive (recalcitrant) seeds, respectively. We compared the seeds of these two species to characterize the developmentally driven changes in the levels of peptide-bound methionine sulfoxide (MetO) and the abundance of methionine sulfoxide reductases (Msrs) B1 and B2, with respect to the cellular redox environment. Protein oxidation at the Met level was dynamic only in Norway maple seeds, and the reduced MsrB2 form was detected only in this species. Cell redox status, characterized by the levels of reduced and oxidized ascorbate, glutathione, and nicotinamide adenine dinucleotide (NAD)/phosphate (NADP), was clearly more reduced in the Norway maple seeds than in the sycamore seeds. Clear correlations between MetO levels, changes in water content and redox status were reported in orthodox *Acer* seeds. The abundance of Msrs was correlated in both species with redox determinants, mainly ascorbate and glutathione. Our data suggest that MsrB2 is associated with the acquisition of desiccation tolerance and that ascorbate might be involved in the redox pathway enabling the regeneration of Msr via intermediates that are not known yet.

## 1. Introduction

Seeds are reproductive structures that evolved to successfully colonize the terrestrial environment by developing important features, including desiccation tolerance and seed dormancy. Seed development begins with fertilized ovules; however, seed morphogenesis, maturation sensu stricto (seed filling) and late seed maturation are the major developmental phases leading to mature embryo formation [1]. Desiccation tolerance is an important physiological trait of seeds, pollen, and resurrection plants. Desiccation tolerance involves the ability to withstand extreme dehydration and to repair damage upon rehydration by activation of protective mechanisms. It is based on the ability of the cell to enter into a quiescent state, fill the vacuole, accumulate protective molecules (sugars, late embryogenesis abundant proteins, heat-shock proteins) and produce antioxidants (reviewed in Dekkers et al. [2]). Orthodox seeds are desiccation-tolerant and thus can withstand desiccation to less than 0.07 g H_2_O g^−1^ dry weight (DW), intermediate seeds survive dehydration to approximately 0.1–0.2 g H_2_O g^−1^, whereas in recalcitrant seeds, which are desiccation-sensitive, the water content (WC) limit ranges from 0.2 to 0.3 g H_2_O g^−1^ DW [3,4,5,6]. Orthodox seeds acquire desiccation tolerance during development, whereas recalcitrant seeds are desiccation-sensitive during development and after shedding [3,7]. Transcriptome, genome and proteome analyses have contributed to deciphering mechanisms of desiccation tolerance in seeds [2,8], and many studies have simultaneously examined the phenomenon of recalcitrance [4,9]. Orthodox and recalcitrant seeds differ in terms of several characteristics, e.g., the structural integrity of membranes and organelles after water removal, metabolic shutdown, the antioxidant system, the availability of protectants including sucrose, oligosaccharides, and late embryogenic abundant proteins [4,10].

Seeds of genetically related species of the *Acer* genus, namely Norway maple (*Acer platanoides* L.) and sycamore (*Acer pseudoplatanus* L.), representing orthodox and recalcitrant types, respectively, became a model to study the differences between such contrasted seeds during developmental transitions [11,12] and during drying and desiccation [13,14,15,16,17]. Both *Acer* species are found in the temperate zone. Despite a small difference in the times of flowering and embryo formation, the DW of their seeds peaks at approximately the same time [18]. During *Acer* seed development, the redox balance is determined by the activity of the ascorbate-glutathione cycle, and concentrations of mainly glutathione are linked to the acquisition of desiccation tolerance [11]. Developing *Acer* seeds also differ in proteins sensitive to cysteine oxidation and abundance of peroxiredoxins (Prxs) [12]. The above studies indicated that molecules determining the cellular redox environment should be of special interest for further exploration when defining differences between orthodox and recalcitrant seeds. Because the distinct levels and redox status of pyridine nucleotides, nicotinamide adenine dinucleotide (NAD) and its phosphorylated form (NADP) especially contribute to the orthodox and recalcitrant phenotype of dried *Acer* seeds [19]. NAD and NADP are crucial coenzymes involved in maintaining the balance between the rates of oxidation and reduction in numerous processes and pathways in plants [20,21], including the plant-specific ascorbate-glutathione cycle [22].

In general, controlled oxidation is a key attribute of the developmental transitions in plants [23], and redox changes are considered seed viability markers [24], and affect many seed traits, including size, dormancy, and sensitivity to aging [23]. Reactive oxygen species (ROS) are dynamically generated in plants (reviewed in Huang et al. [25]), and until their removal, ROS oxidize molecules including proteins. Oxidation of thiol-containing cysteine, sulfur-containing methionine (Met) and nitration of tyrosine (Tyr) are reversible posttranslational modifications considered to participate in redox signaling [26]. Met oxidized to methionine sulfoxide (MetO) is reduced back to Met enzymatically by methionine sulfoxide reductases (Msrs). The (S)- and (R)-stereoisomers of protein-bound MetO are reduced by specific A-type (MsrA) and B-type (MsrB) enzymes, respectively, which are present in many isoforms [27,28,29]. For example, 14 *Msr* genes, five *MsrA* isoforms and nine *MsrB* isoforms are encoded by the Arabidopsis genome [30,31]. *MsrB* genes encode proteins with masses of approximately 15 kDa. Recently, the accumulation of MsrB was found to be related to the reestablishment of desiccation tolerance in germinating seeds [32], and MsrB2 abundance monitored in gradually drying Norway maple seeds was assumed as an important player of the redox network associated with acquisition of desiccation tolerance [17].

Msrs play protective and signaling roles under many abiotic and biotic stresses (reviewed in Rey and Tarrago [33]). Based on the cis-regulatory elements found in the promoters of *Msr* genes from Arabidopsis, poplar and rice [30,31], Msrs putatively participate in the regulation of plant growth, including seed development [34]. Experimental studies indicated that plastidial Msrs B1 and B2 contribute to seed longevity [35]. The removal of MetO results in the oxidation of Msrs, which requires thioredoxins and NADPH thioredoxin reductase (TrxR) as electron source in cytosol. In plastids, the reducing power originates from the photosynthetic electron transfer chain via thioredoxins. To function as MetO reductases, Msrs require efficient regeneration to switch between oxidized and reduced forms. MsrB2 and other 2-Cys Msrs are regenerated through a thioredoxin (Trx)-dependent mechanism, whereas MsrB1 regeneration uses glutaredoxin (Grx)- and GSH/Grx-dependent mechanisms [36,37]. In this context, the potential reductants of plastidial Msrs remain unknown in seeds, where photosynthesis and thus the Trx-dependent Msr reduction system are probably not active during the maturation phase.

Orthodox and recalcitrant seeds have markedly differing longevities [4], which encouraged us to investigate whether plastidial MsrB isoforms participate in redox regulation during seed development in Norway maple and sycamore. We report that developing Norway maple and sycamore seeds have differing dynamics in terms of MetO levels and MsrB abundance. In addition, the reducing power, which was assessed by pools of reduced and oxidized ascorbate, glutathione, and NAD(P), varied between the two species. Correlation matrixes indicated that ascorbic acid (AsA), GSH and pyridine nucleotides likely drive in concert the redox machinery more efficiently in Norway maple than in sycamore and that AsA could participate in the regeneration of plastidial Msr activity in the context of seed physiology.

## 2. Materials and Methods

### 2.1. Plant Material

Developing seeds of Norway maple and sycamore were used for the study. The seeds were harvested at weekly intervals, starting from the 12th to 23rd weeks after flowering (WAF) (Norway maple) and from the 11th to 22nd WAF (sycamore). The material was collected from single trees localized in Kórnik, western Poland. Water content (WC) measurements were made each harvest day using triplicates of 10 embryonic axes and 5 cotyledons by drying at 105 °C for 24 h. The same day, the remaining seeds were removed from the seed coat and then weighed and frozen at −80 °C in samples of 20 embryonic axes and 5 cotyledons for further analyses.

### 2.2. Protein Extraction

Samples (20 embryonic axes and 5 cotyledons) were ground with liquid nitrogen in a chilled mortar and pestle. The dry powder was incubated with a 50 mM phosphate buffer (pH 7.0) and with 2% polyvinylpolypyrrolidone at 4 °C for one hour, shaking every 15 min. We applied 300 µL of the buffer for each 0.15 g of seed tissue. The supernatant was collected after centrifugation for 20 min at 20,000× *g* at 4 °C. The protein content was determined by the Bradford method [38].

### 2.3. Determination of MetO

Determinations of MetO, Met, Tyr, and tryptophan (Trp) were performed according to the method described by Baxter et al. [39] using an Agilent Infinity II 1260 model HPLC system (Agilent Technologies, Wilmington, DE, USA) equipped with an Agilent Poroshell 120 Stablebond Aq (3.0 × 150 mm, 2.7 µm) particle column heated to 40 °C and mobile phases based on ultra-pure water (A) and 0.02 M monopotassium phosphate buffer (pH 2.9) combined with 17.5% acetonitrile and 17.5% isopropanol (B) in conjunction with an Agilent 1260 Infinity II Diode Array Detector. Proteins isolated in PIPES buffer were digested (20 h/37 °C) using a combination of three enzymes: pronase, leucine aminopeptidase, and prolidase which were obtained from Sigma–Aldrich (St. Louis, MO, USA). The detection wavelengths were 214 nm (for MetO and Met) and 280 nm (for Tyr and Trp), with references at 590 nm (Supplementary Materials Figure S1). The only modifications to the method were adjustments to the model of chromatograph and column. Thus, the elution program was 0% B from 0.0 to 5.0 min (flow rate of 0.15 mL min^−1^), 0 to 16% B from 5.0 to 8.0 min (flow rate of 0.3 mL min^−1^), 16 to 100% B from 8.0 to 16.0 min (flow rate of 0.3 mL min^−1^), and 0% B from 16.0 to 18.0 min (flow rate from 0.3 to 0.15 mL min^−1^). The MetO ratio was calculated in relation to the total pool of Met detected on the basis of calibration curves.

### 2.4. Western Blot Analysis

Samples containing 15 µg of protein calculated based on the calibration curve data representing the linear increase between the protein concentration and band intensity were loaded onto a 12% SDS-PAGE gel. The PageRuler Prestained Protein Ladder (Thermo Scientific) was used as the mass marker (Supplementary Materials Figure S2). SDS-PAGE electrophoresis was carried out using the Mini-PROTEAN^®^ Tetra Cell (Bio-Rad Laboratories, Inc., Grand Junction, CO, USA). Separated proteins were transferred to a polyvinylidene fluoride membrane (PVDF, Immobilon-P, Millipore) using a mini trans-Blot^®^ Cell (Bio-Rad) under 350 mA for 1 h. The PVDF was blocked in 5% skimmed milk and incubated overnight in primary antibodies (1:1000 dilution) raised against AtMsrB1 and AtMsrB2 [40]. Secondary antibodies were conjugated with alkaline phosphatase (AP, Sigma-Aldrich St. Louis, MO, USA). Protein detection was performed colorimetrically with identical exposure time using 5-bromo-4-chloro-3-indolyl phosphate (Sigma-Aldrich) and tetrazolium blue nitrate (NBT; Sigma-Aldrich) as the AP substrate. Western blot images were analyzed densitometrically in triplicate using the UviBand (UviTec, Cambridge, United Kingdom) program in the Fire Reader Gel Documentation System. Band density was calculated based on the volume (V) of the band as the sum of all 3D intensities (I) coded on a scale of 256 gray levels. The data are presented in relative units obtained from V = Σn_i_I and the number of pixels inside the area of the band.

### 2.5. Immunolocalization

Cotyledons and embryonic axes were fixed in a mixture of 2% glutaraldehyde (pH 6.8; Polysciences, Warrington, USA) and 2% (*v*/*v*) formaldehyde (pH 6.8; Polysciences) for 12 h 4 °C. Then, the material was rinsed three times in 1x PBS (Sigma-Aldrich) buffer. The samples were sectioned (30 µm) using a vibratome Leica VT 1200S (Leica Biosystems). MsrB1 and MsrB2 were localized using primary rabbit antibodies against MsrB1 and MsrB2 at a dilution of 1:100. The tyramide signal amplification (TSA) technique (ThermoFisher Scientific, Carlsbad, CA, USA) was applied because its detection sensitivity is more than 1000 times higher than that of the standard immunolocalization protocol. All steps of the reaction were performed as described by Wojciechowska et al. [41]. The localization results were analyzed and documented with a Leica TCS SP5 confocal microscope (Leica) using an argon laser emitting light at a wavelength of 488 for Alexa Fluor 488. Two negative controls were performed: (1) omitting the primary antibody and (2) omitting antibodies conjugated with HRP. Reactions were carried out on cotyledons and embryonic axes.

### 2.6. Determination of NADPH-Dependent Reductases

Determination of NADPH-dependent reductases was based on the reduction of 5,5′-dithiobis(2-nitrobenzoic) acid (DTNB) with NADPH to 2-nitro-5-thiobenzoate, which produces a strong yellow color that is measured at 412 nm, as described by Alipour et al. [19]. The reaction mixture contained reaction buffer consisting of 50 mM phosphate buffer pH 7.0, 50 mM KCl, 1 mM ethylenediaminetetraacetic acid (EDTA), 1 mM DTNB, protein extract and 8 mM NADPH. Simultaneously the reaction mixture lacking NADPH was measured to eliminate the reduction of DTNB by thiol-containing components of the measured extract. The reaction was measured for 3 min using an Infinite M200 PRO (Tecan, Männedorf, Switzerland) plate reader and Magellan software.

### 2.7. Determination of Ascorbate, Glutathione and NAD(P) Levels

The levels of ascorbate, glutathione and NAD(P) were measured according to the method described by Queval and Noctor [42]. Seed samples were ground in 1 mL 0.2 N HCl and centrifuged for 10 min at 4 °C and 14,000 rpm. The extract intended for ascorbate and glutathione determination (500 µL) was neutralized and the pH was adjusted to a 4.5–5 range. The extract intended for NAD(P) determination (200 µL) was incubated at 100 °C for 2 min and neutralized to pH 6–7 after cooling. The Infinite M200 PRO (Tecan) plate reader and Magellan software were used for all measurements.

For ascorbate determination, the assay was adapted from the methods of Hewitt and Dickes [43] and Queval and Noctor [42] to measure extremely low AsA quantities in drying seeds, and this method is described by Wojciechowska et al. [17]. AsA was measured in neutralized extracts by its ability to absorb light at 265 nm in a slightly acidic environment. Asc, referred to as “total ascorbate”, was measured after conversion of DHA to AsA by incubation with 25 mM dithiothreitol at pH 4.7. The measurements were performed in 0.1 mM acetic acetate buffer containing 5 mM EDTA. DHA in the assays was determined by subtracting free AsA from the total AsA.

For determination of glutathione, the neutralized extract was treated with 2-vinylpyridine (2-VP) for 30 min at RT and centrifuged twice for 15 min at 4 °C and 14,000× *g*. The reaction mixture contained 120 mM NaH_2_PO_4_/10 mM EDTA pH 7.5, 12 mM DTNB, 10 mM NADPH, MQ water and extract (to measure total glutathione, GSH + GSSG) or 2-VP treated extract (to measure GSSG), and glutathione reductase (0.2 U). The kinetic measurements were performed at 412 nm. Calculations were based on calibration curves prepared using GSSG and GSH (Sigma-Aldrich) as standards. The degree of oxidation (DO) of glutathione was calculated according to Meyer and Hell [44].

For determination of NAD(P) pools, the reaction mixture consisted of 10 mM HEPES/2 mM EDTA (pH 7.5), 1.2 mM 2,6-dichlorophenolindophenol, 10 mM phenazine methosulfate, and neutralized extracts. Glucose-6-phosphate dehydrogenase (2 U) and 10 mM glucose-6-phosphate were added to measure NADP. Alcohol dehydrogenase (25 U) and ethanol were added to measure NAD. The kinetic measurements were performed at 340 nm. The levels of reduced and oxidized forms of NAD(P) were calculated based on calibration curves prepared using NADPH, NADP^+^, NADH, and NAD^+^ (Sigma-Aldrich) as standards.

### 2.8. Statistical Analyses

Data are the means of three independent replicates ± the standard deviation (STD) or standard error (SE). Statistically significant differences are indicated with different letters (one-way ANOVA, followed by Tukey’s test at *p* ≤ 0.05). The relationships between particular parameters were examined using Pearson’s correlation coefficient analysis. Proportional data were transformed prior to analysis using the arcsine transformation. R statistical software was used to calculate Pearson’s correlation [45]. Correlation matrixes were made using the corrplot package [46].

## 3. Results

### 3.1. Changes in Seed Hydration Levels

**Figure 1:**
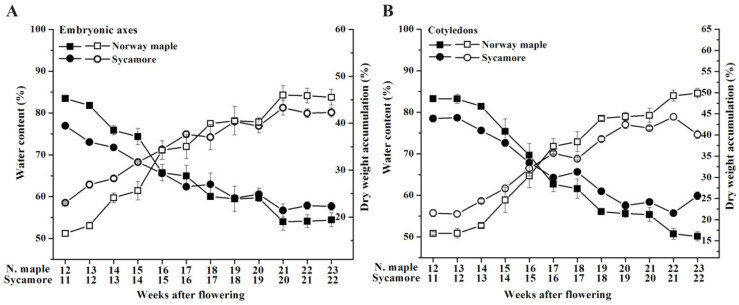
Changes in water content (solid symbol) and dry mass accumulation (open symbol) in the embryonic axes (**A**) and cotyledons (**B**) of developing seeds of Norway maple (squares) and sycamore (circles) collected during the 11th–23rd weeks after flowering. Data are the means of three independent replicates ± the standard deviation.

Seed development was analyzed from the 12th to 23rd WAF in Norway maple and from the 11th to 22nd WAF in sycamore. The WC gradually decreased as seeds of both species matured (Figure 1). At the beginning of development, the embryonic axes and cotyledons of Norway maple were more hydrated than those of sycamore. In contrast, at the end of maturation, the level of hydration in Norway maple seeds was lower than that in sycamore seeds. Thus, 12 weeks of seed development and maturation resulted in WC decreases of ~29% and ~19% in the embryonic axes of Norway maple and sycamore, respectively (Figure 1A). In the cotyledons of sycamore, the loss of water reached ~23%, compared to ~33% in the cotyledons of Norway maple (Figure 1B). The changes in seed hydration were reflected in the changes in DW in both the embryonic axes (Figure 1A) and cotyledons (Figure 1B).

### 3.2. Protein and Peptide-Bound MetO Content

**Figure 2:**
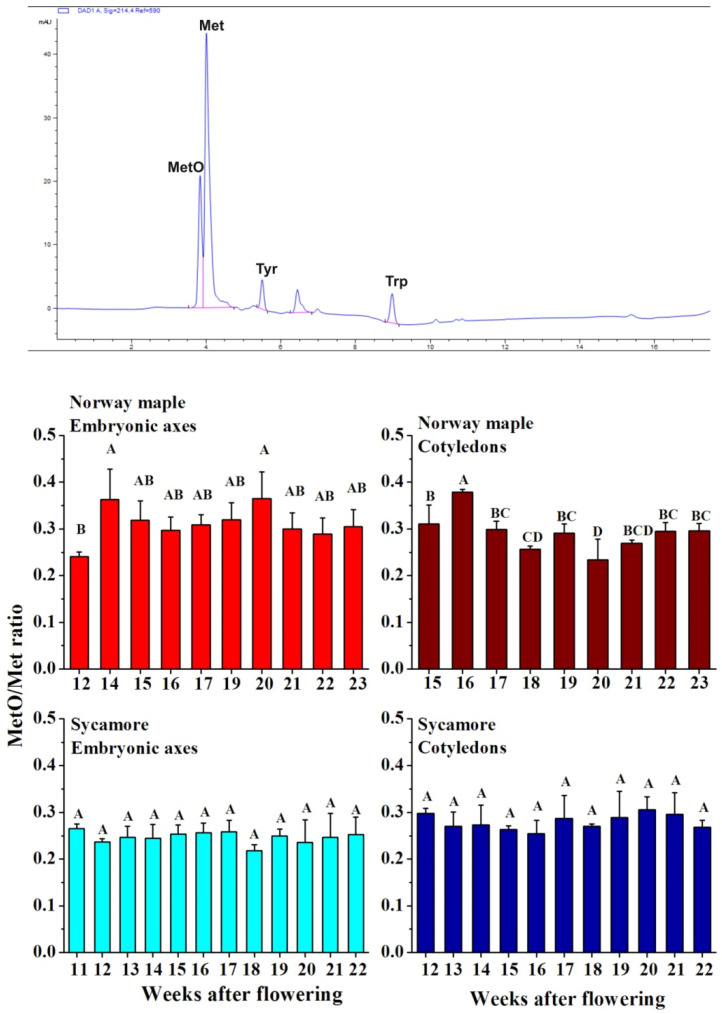
Detection of methionine (Met) oxidized to methionine sulfoxide (MetO) in proteins of developing Norway maple and sycamore seeds collected during the 11th–23rd weeks after flowering. A representative chromatogram indicating peaks recognized at 214 nm is given. Standards of Met, MetO, tyrosine (Tyr) and tryptophan (Trp) were used for the calibration curves. The MetO ratio was calculated in relation to the total pool of methionine. Data are the means of three independent replicates ± the standard deviation. Identical letters indicate groups not significantly differentiated according to Tukey’s test.

The level of soluble proteins increased as maturation progressed, particularly in cotyledons (Supplementary Materials Figure S3). After the 17th WAF, protein abundance increased up to 80 mg per g DW during maturation. Sycamore cotyledons accumulated significantly more proteins than Norway maple cotyledons. Similarly, the amount of proteins was higher in the sycamore embryonic axes than in the Norway maple embryonic axes. The peptide-bound MetO levels did not change in developing sycamore seeds, whereas in Norway maple seeds, significant changes were reported (Figure 2). The highest MetO level was detected at the 14th and 20th WAF in embryonic axes and at the 16th WAF in cotyledons. Interestingly, the developmental stage with the greatest difference between the MetO levels in the embryonic axes and cotyledons corresponded to the 20th WAF (Figure 2).

### 3.3. Abundance of MsrB1 and MsrB2 Proteins

**Figure 3:**
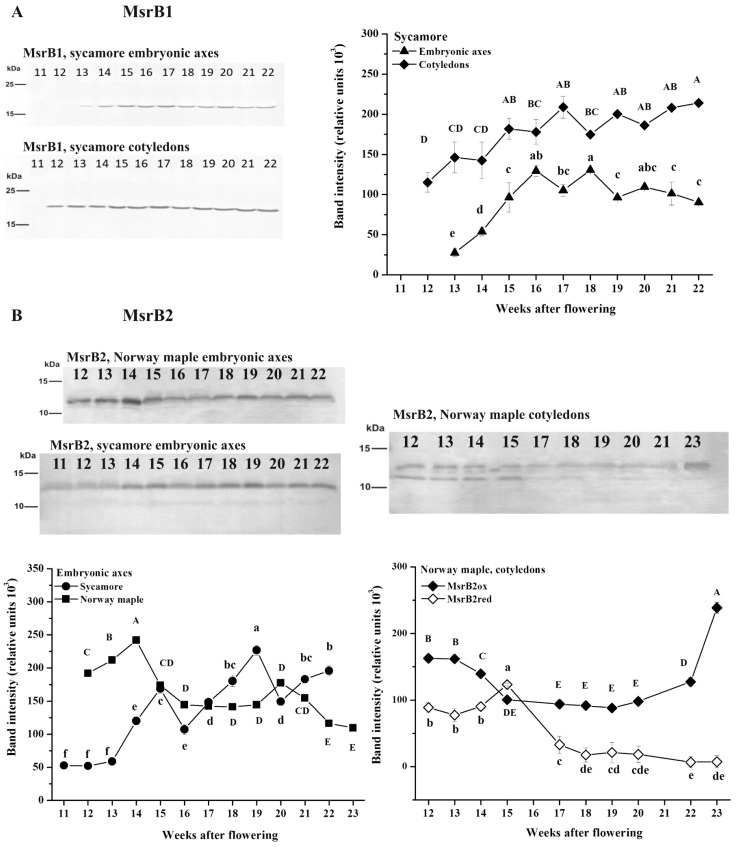
Immunodetection of MsrB1 (**A**) and MsrB2 (**B**) proteins in embryonic axes and cotyledons of Norway maple and sycamore seeds collected during the 11th–23rd weeks after flowering. Representative Western blots are given. Samples containing 15 µg of protein were loaded per lane. Antibodies against AtMsrB1 and AtMsrB2 were used. The molecular mass was estimated based on PageRuler Prestained Protein Ladder (Thermo Scientific). Identical letters indicate groups not significantly different according to Tukey’s test.

The abundance of the two plastidial B-type MSR isoforms, MsrB1 and MsrB2, was investigated in *Acer* seeds (Figure 3). MsrB1 was detected uniquely in sycamore as a 17 kDa protein (Figure S4). In embryonic axes, detectable amounts were revealed at the 13th WAF, whereas in cotyledons, MsrB1 was detectable one week earlier, and the protein was present in larger amounts throughout the whole development and during the maturation phase (Figure 3A). Interestingly, a linear increase in the amount of MsrB1 was observed at the 12th–17th WAF in cotyledons. In embryonic axes, an increased abundance of MsrB1 was detected at the 15th–17th WAF. MsrB2 was detectable in seeds of both *Acer* species. In sycamore, MsrB2 was detectable uniquely in the embryonic axes as a one-protein band with a molecular mass of 14 kDa (Figure 3B, Supplementary Materials Figure S4).

**Figure 4:**
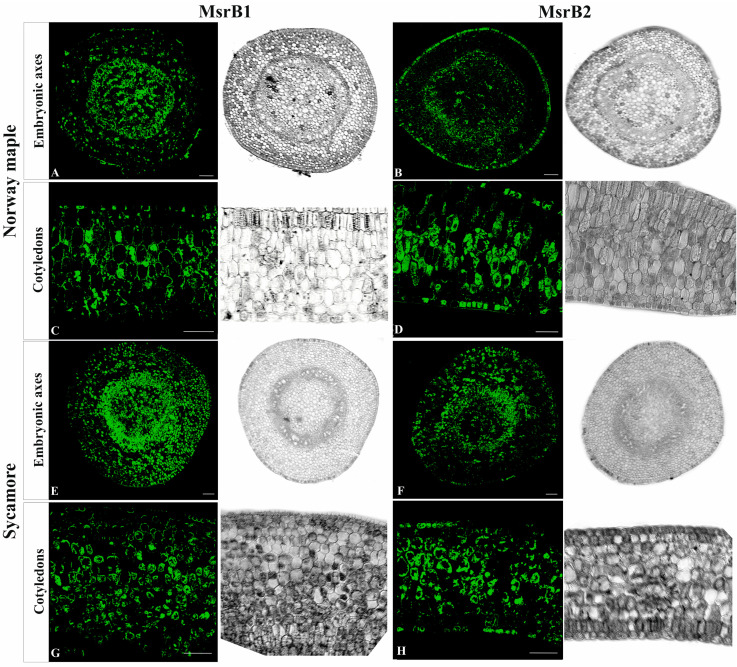
Immunohistochemical localization (green fluorescence) of MsrB1 and MsrB2 proteins in the embryonic axes (**A**,**B**) and cotyledons (**C**,**D**) of mature Norway maple seeds and in the embryonic axes (**E**,**F**) and cotyledons (**G**,**H**) of mature sycamore seeds. Bars = 100 µm.

In Norway maple, this protein was present in both the embryonic axes and cotyledons and was present in two different redox forms with molecular masses of 12 and 13 kDa [40]. A greater amount of MsrB2 was revealed in embryonic axes at the 14th WAF. The two redox MsrB2 forms were revealed in cotyledons at the 12th–16th WAF. During later stages, the 12 kDa form was hardly detectable in the cotyledons, and the protein abundance decreased. In embryonic axes, higher amounts of MsrB2 were noticed in Norway maple than in sycamore. Immunohistochemistry was performed to detect whether the MsrB1 protein might be expressed at levels undetectable by the Western blot method. Using confocal laser scanning microscopy and signal enhancement, both MsrB proteins were detected within embryonic axes and cotyledons of both species (Figure 4). In most organs, MsrB2 was found more abundant than MsrB1 [30,40]. According to the Protein Abundance Database (PaxDb) 4.1, in *Arabidopsis thaliana*, the whole-organism MsrB1 protein abundance, expressed in parts per million (ppm), is four orders of magnitude lower than that of the whole-organism MsrB2 abundance. This fact might explain why MsrB1 was undetectable using standard Western blot analysis (Figure 3A) and detectable using the TSA signal enhancer in confocal microscopy (Figure 4).

**Figure 5:**
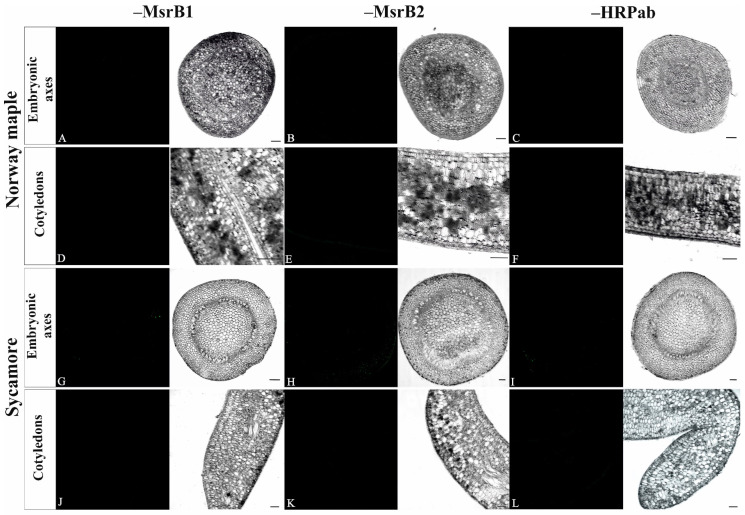
Negative controls for immunohistochemical reactions of MsrB1 and MsrB2 localization, performed on embryonic axes and cotyledons of Norway maple and sycamore seeds. The reactions were performed omitting: the primary antibody MsrB1 (**A**,**D**,**G**,**J**), the primary antibody MsrB2 (**B**,**E**,**H**,**K**), and the secondary antibody HRPab conjugated with horseradish peroxidase (**C**,**F**,**I**,**L**). For each reaction image an corresponding image in bright light is given.

Two negative controls were performed: (1) omitting the primary antibody and (2) omitting antibodies conjugated with HRP. Reactions carried out on embryonic axes and cotyledons of both species confirmed specificity of primary antibodies (Figure 5).

### 3.4. Activity of NADPH-Dependent Reductases

**Figure 6:**
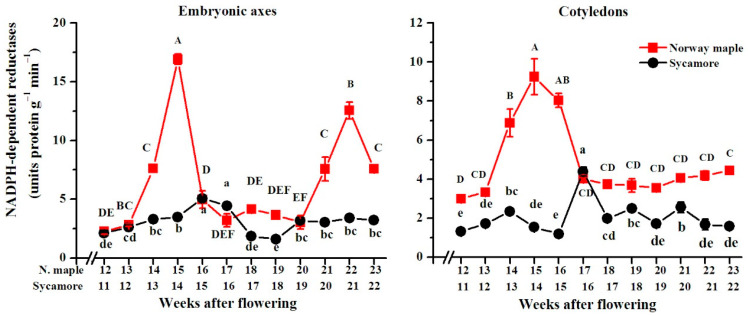
Changes in the activity of total NADPH-dependent reductases reported in the embryonic axes and cotyledons of developing seeds of Norway maple (squares) and sycamore (circles) collected during the 11th–23rd weeks after flowering. Data are the means of three independent replicates ± the standard error. Statistically significant differences are indicated with different letters (one-way ANOVA, followed by Tukey’s test at *p* < 0.05). The capital letters refer to Norway maple.

The activity of NADPH-dependent reductases was higher in Norway maple seeds than in sycamore seeds (Figure 6). In Norway maple, the highest activity was reported at the 15th WAF in both embryonic axes and cotyledons, although this peak was nearly two-fold lower in cotyledons than in embryonic axes. In sycamore, the peak in activity of NADPH-dependent reductases was also detected in embryonic axes at the 15th WAF but was 3-fold lower than the peak observed in Norway maple. In the sycamore cotyledons, the highest activity was measured at the 16th WAF and was two-fold lower than the highest activity in the cotyledons of Norway maple.

### 3.5. NAD(P) Content

**Figure 7:**
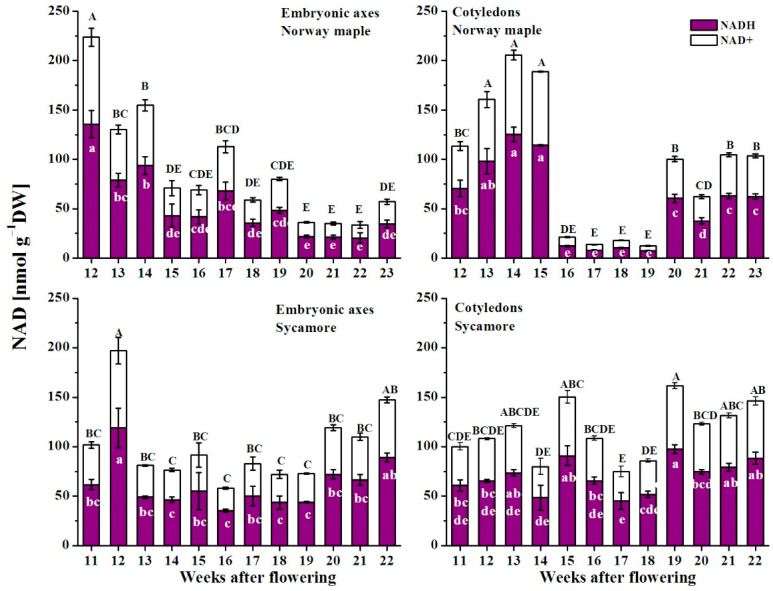
Changes in the levels of the reduced (NADH) and oxidized (NAD^+^) forms of nicotinamide adenine dinucleotide (NAD) in the embryonic axes and cotyledons of developing seeds of Norway maple and sycamore collected during the 11th–23rd weeks after flowering. Data are the means of three independent replicates ± the standard error. Statistically significant differences are indicated with letters (one-way ANOVA, followed by Tukey’s test at *p* < 0.05). The capital letters refer to NAD^+^.

The pool of NAD in the embryonic axes of Norway maple seeds was the highest at the morphogenesis stage, subsequently decreased, peaked at the 17th WAF and reached the lowest values at the end of maturation at the 20th–23rd WAF (Figure 7). The average concentrations of NAD in cotyledons were comparable to those in embryonic axes at the 12th–15th WAF, and then markedly decreased up to ten times, reaching the lowest values at the 16th–19th WAF. Then, the NAD concentration increased five times and remained nearly constant to the end of maturation. The NAD concentration in the embryonic axes of sycamore seeds did not vary significantly, except for at the 12th WAF, when the pool of NAD doubled and increased until the 22nd WAF at the end of maturation. In sycamore cotyledons, the NAD concentration remained at approximately 125 nmol g^−1^ DW, except for at the 15th and 19th WAF, when maximal values were detected, and except for the period between the two peaks, i.e., at the 16th–18th WAF, when significantly low NAD concentrations were measured.

**Figure 8:**
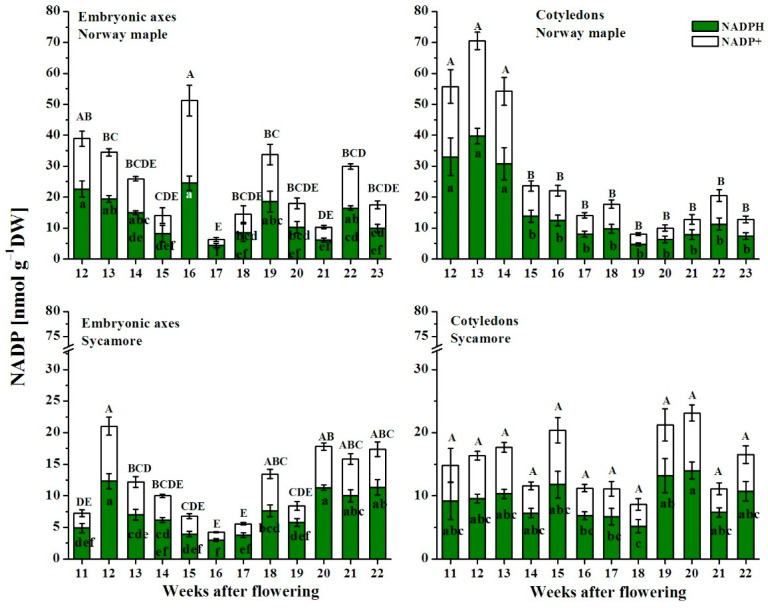
Changes in the levels of the reduced (NADPH) and oxidized (NADP^+^) forms of nicotinamide adenine dinucleotide phosphate (NADP) in the embryonic axes and cotyledons of the developing seeds of Norway maple and sycamore collected during the 11th–23rd weeks after flowering. Data are the means of three independent replicates ± the standard error. Statistically significant differences are indicated with different letters (one-way ANOVA, followed by Tukey’s test at *p* < 0.05). The capital letters refer to NADP^+^.

The NADP pool was up to three times higher in Norway maple seeds than in sycamore seeds (Figure 8). Three clear peaks of NADP were reported in Norway maple embryonic axes at the 16th, 19th and 21st WAF, whereas in cotyledons, the NADP level was relatively stable at the first morphogenesis stages, exhibiting concentrations approximately three times higher than those during the maturation stages. The highest concentration of NADP in the sycamore embryonic axes was observed at the 12th WAF, after which the NADP concentration decreased until the 16th WAF and then peaked again at the 18th WAF to a level that was sustained to the end of maturation. The changes in NADP concentration in sycamore cotyledons globally reflected those in NAD concentration.

**Figure 9:**
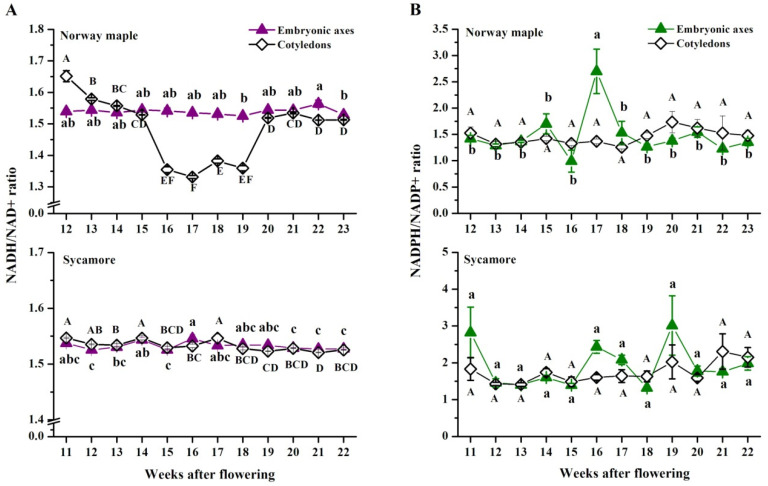
Changes in the ratios of (**A**) reduced (NADH) to oxidized (NAD^+^) forms of nicotinamide adenine dinucleotide (NAD) and (**B**) reduced (NADPH) to oxidized (NADP^+^) forms of nicotinamide adenine dinucleotide phosphate (NADP) in the embryonic axes and cotyledons of the developing seeds of Norway maple and sycamore collected during the 11th–23rd weeks after flowering. Data are the means of three independent replicates ± the standard error. Statistically significant differences are indicated with different letters (one-way ANOVA, followed by Tukey’s test at *p* < 0.05). The capital letters refer to the embryonic axes.

The NADH/NAD^+^ ratio (Figure 9A) in the embryonic axes of Norway maple was stable during the whole analyzed period, reaching ~1.5, whereas in cotyledons, the highest ratio was detected at the 12th WAF. A significantly decreased NADH/NAD^+^ ratio occurred in the four following weeks between the 16th and 19th WAF. Afterward, the ratio returned to its original level. The NADH/NAD^+^ ratio displayed discrete changes in both the embryonic axes and cotyledons of sycamore. The NAD(P)H/NAD(P)^+^ ratios in both *Acer* species were higher than 1 (Figure 9). Sycamore cotyledons were characterized by a slightly higher NADPH/NADP^+^ ratio than Norway maple cotyledons (Figure 9B). The NADPH/NADP^+^ ratio reported in the embryonic axes of Norway maple was quite stable until the 15th WAF, decreased slightly to ~1.0 at the 16th WAF, and subsequently increased to ~2.7 at the 17th WAF. Afterward, the ratio decreased to ~1.5 and remained stable until the 23rd WAF. The NADPH/NADP^+^ ratio measured in the sycamore embryonic axes was quite stable; however, two peaks were noticed at the 11th and 19th WAF when the ratio reached 3.0.

### 3.6. Ascorbate Content

**Figure 10:**
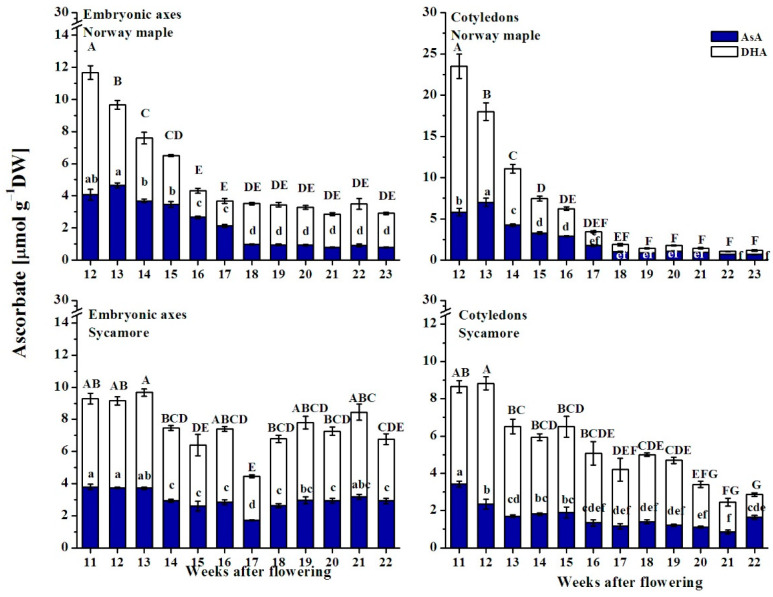
Changes in the levels of the reduced (AsA) and oxidized (DHA) forms of ascorbate reported in the embryonic axes and cotyledons of the developing seeds of Norway maple and sycamore collected during the 11th–23rd weeks after flowering. Data are the means of three independent replicates ± the standard error. Statistically significant differences are indicated with different letters (one-way ANOVA, followed by Tukey’s test at *p* < 0.05). The capital letters refer to DHA.

The Asc pool, which includes reduced ascorbate (AsA) and DHA, the oxidized form of AsA, decreased approximately four times in the embryonic axes and approximately ten times in the cotyledons of Norway maple during the studied developmental period, whereas in sycamore seeds, the Asc pool was halved in cotyledons and only slightly reduced in embryonic axes (Figure 10). The concentration of Asc clearly decreased during Norway maple seed development until the 18th WAF, and then remained at a stable level, which was higher in embryonic axes than in cotyledons. DHA was predominant, particularly in embryonic axes, in the last stages of Norway maple seed development, whereas in sycamore seeds, DHA was more abundant than AsA during the whole developmental period.

**Figure 11:**
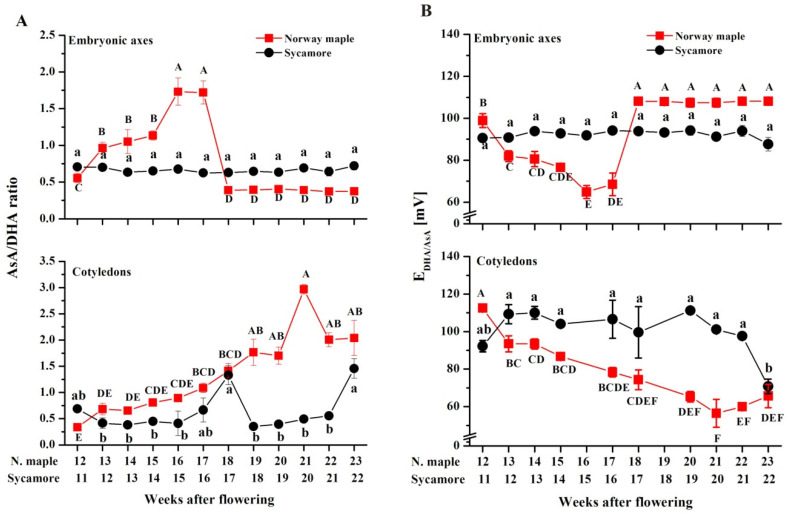
Changes in the ratios of reduced (AsA) to oxidized (DHA) forms of ascorbate (**A**) and half-cell reduction potential (E_DHA/AsA_) of ascorbate (**B**) in the embryonic axes and cotyledons of developing seeds of Norway maple (squares) and sycamore (circles) collected during the 11th–23rd weeks after flowering. Data are the means of three independent replicates ± the standard error. Statistically significant differences are indicated with different letters (one-way ANOVA, followed by Tukey’s test at *p* < 0.05). The capital letters refer to Norway maple.

There was a clear difference in the AsA/DHA ratio between Norway maple and sycamore embryonic axes (Figure 11A). A relatively unchanged AsA/DHA ratio of 0.75 was reported in sycamore, whereas in Norway maple, the AsA/DHA ratio increased until it reached a value of approximately two at the 16–17th WAF, after which it substantially decreased and remained unchanged until the end of maturation. The cotyledons of Norway maple and sycamore differed markedly between the 18/19th and 21st/22nd WAF. At this time, the AsA/DHA ratio was remarkably high in Norway maple cotyledons and peaked at three, whereas in sycamore, the ratio did not exceed 0.5.

Changes in concentrations of Asc reduced and oxidized forms, were reflected by the half-cell reduction potential (E_DHA/AsA_). The lower the E_DHA/AsA_, the higher the AsA content in the Asc pool (Figure 11B). A clear redox change from highly reduced to highly oxidized was reported uniquely in the embryonic axes of Norway maple seeds at the 17/18th WAF. Interestingly, in cotyledons, E_DHA/AsA_ was substantially lower in most analyzed developmental stages in Norway maple than in sycamore.

### 3.7. Glutathione Content

**Figure 12:**
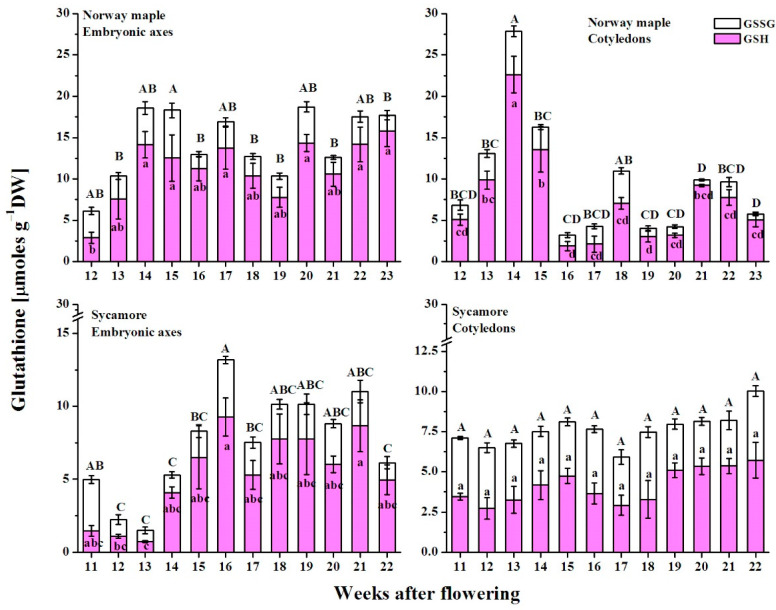
Changes in the levels of the reduced (GSH) and oxidized (GSSG) forms of glutathione reported in the embryonic axes and cotyledons of developing seeds of Norway maple and sycamore collected during the 11th–23rd weeks after flowering. Data are the means of three independent replicates ± the standard error. Statistically significant differences are indicated with different letters (one-way ANOVA, followed by Tukey’s test at *p* < 0.05). The capital letters refer to GSSG.

During Norway maple seed development, the maximal glutathione concentrations were in the range of 20 and 30 µmoles g^−1^ DW in embryonic axes and cotyledons, respectively, whereas in sycamore they were in the range of 12 and 10 µmoles g^−1^ DW, respectively (Figure 12). In both tissues of Norway maple, the total glutathione pool increased at the morphogenesis phase and peaked as maturation started at the 14th WAF. Then, the total glutathione level increased again at the 17th and 20th WAFs in embryonic axes and one week later at the 18th and 21st WAFs in cotyledons. In sycamore embryonic axes, the glutathione content increased until the 16th WAF, and it remained relatively unchanged until the end of maturation. Notably, the average glutathione concentration in embryonic axes was half compared to that in Norway maple. In sycamore cotyledons, this level was relatively stable throughout seed development for both the reduced and oxidized forms.

**Figure 13:**
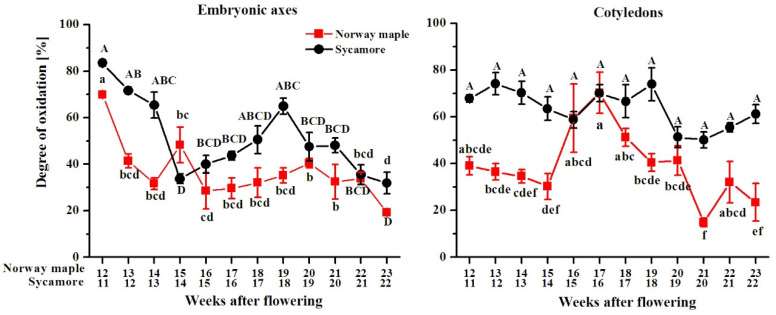
Changes in the degree of oxidation of glutathione in the embryonic axes and cotyledons of developing seeds of Norway maple (squares) and sycamore (circles) collected during the 11th–23rd weeks after flowering. Data are the means of three independent replicates ± the standard error. Statistically significant differences are indicated with different letters (one-way ANOVA, followed by Tukey’s test at *p* < 0.05). The capital letters refer to sycamore.

Glutathione exists in reduced (GSH) and oxidized (GSSG) states. The glutathione degree of oxidation (DO) reported in Norway maple embryonic axes was the highest at the morphogenesis stage, after which it decreased and remained relatively unchanged during seed maturation at a level of approximately 30% (Figure 13). Sycamore embryonic axes exhibited higher DO values than those in Norway maple embryonic axes, except for at the 14th WAF, with clear change trends. The DO values decreased until the 14th WAF, increased and peaked at the 18th WAF, after which they decreased again, reaching the lowest DO at the end of maturation. The average DO in sycamore cotyledons was approximately 60%, whereas a value as high as 60% was reported in Norway maple cotyledons only at the 16–18th WAF. Except during this period, the DO values in the cotyledons of Norway maple were 40% and decreased to approximately 20%. The above data clearly indicate that in both embryonic axes and cotyledons, glutathione is more abundant and more reduced in Norway maple than in sycamore.

### 3.8. Correlation Analysis

A correlation coefficient analysis between all measured parameters was performed (Supplementary Materials Figure S5). In Norway maple seeds, the MetO level was positively correlated with changes in the WC and with the activity of NADPH-dependent reductases and ascorbate, but negatively correlated with the protein content and the AsA/DHA ratio. Few links were found in sycamore seeds because only the MetO level was negatively correlated with the NADP^+^ level. The levels of both MsrB1 and MsrB2 in sycamore were negatively correlated with the WC, protein content, AsA and Asc levels, GSH and total glutathione levels. Additionally, the MsrB1 level was correlated with the GSSG level, whereas the MsrB2 level was linked to the DHA content. The MsrB2 abundance in Norway maple was positively correlated with the WC, NADPH and NADP levels, as well as with the AsA, DHA, Asc levels, in both embryonic axes and cotyledons. The amount of the MsrB2 redox form with a low molecular mass was correlated with changes in NADH and NAD^+^ concentrations in embryonic axes and with the levels of GSH and GSSG. In contrast, no such relation were noticed for the MsrB2 redox form with a high molecular mass.

## 4. Discussion

### 4.1. MetO Content in Acer Developing Seeds

Norway maple and sycamore seeds provides a good model for the comparison of seeds from the same genus with contrasted physiology [11,12,47,48]. Here, we used this model for investigating redox biology in developing *Acer* seeds. *Acer* seeds gradually lose water throughout the process of maturation [11,12]. Here, we observed a decrease in seed hydration level from approximately 80% to approximately 50% (Figure 1). Interestingly, the progressive WC decrease was positively correlated with the levels of peptide-bound MetO in Norway maple cotyledons. MetO content depends on efficient ROS removal and proper Msr activity [49,50,51]. ROS acts as signaling molecules during physiological transitions in seeds [52], and H_2_O_2_ peak levels coincide with the acquisition of desiccation tolerance in Norway maple seeds [12]. Among ROS, H_2_O_2_ oxidizes Met to MetO at a slow rate compared to hydroxyl radicals, which are the most powerful oxidizers of Met [53]. The above data emphasize that the discrete changes in the Met redox status of Norway maple seeds might result from posttranslational modifications that function in signaling pathways involved in the acquisition of desiccation tolerance. Consistently, the MetO level clearly decreased upon desiccation in Norway maple seeds, particularly in the embryonic axes, whereas no change was observed in gradually dehydrating sycamore seeds [17]. Compared with cotyledons, embryonic axes are constitutively protected [54] and exhibit a more active antioxidant system during seed development and drying [11,55]. Thus, seed embryonic axes display much more intense redox changes than do cotyledons, and in *Acer* species, this phenomenon is likely more pronounced in Norway maple.

### 4.2. Role of Plastidial MsrBs in Acer Developing Seeds

MsrB1 and MsrB2 proteins are highly expressed in photosynthetic Arabidopsis organs [40], and microarray analyses have since confirmed the presence of *MsrB2* and *MsrB6* transcripts in seeds. Châtelain et al. [35] reported that *MsrB1* and *MsrB2* are expressed in *Medicago truncatula* seeds. Then, Wojciechowska et al. [17] demonstrated that MsrB2 is present in dried Norway maple and sycamore seeds and that MsrB1 is present only in sycamore seeds, as shown here (Figure 2 and Figure 3). The molecular masses of Arabidopsis MsrB1 and MsrB2 are 17 and 15 kDa, respectively [40]. In *Acer* seeds, one unique MsrB1 form was revealed, but two approximately 13 and 14 kDa MsrB2 bands were detected (Figure 3B). Western blot analyses of Arabidopsis MsrB2 revealed two distinct bands with higher and lower molecular masses corresponding to oxidized and reduced forms, respectively [40]. In this context, the two bands detected in Norway maple (Figure 3B) probably correspond to oxidized and reduced forms of MsrB2, henceforth referred to as MsrB2_ox_ and MsrB2_red_ for the upper and lower bands, respectively. In Arabidopsis, the oxidized form of MsrB2 is predominant in the leaves [40]. In Norway maple, MsrB2_ox_ was present throughout seed development, whereas MsrB2_red_ was almost undetectable from the 16th WAF (Figure 3B), indicating a more oxidative environment at the end of maturation. The MsrB2 protein abundance and redox pattern in Norway maple seeds were very different from those in sycamore. In this context, we suggest that MsrB2 might be a new component involved in the redox control of the acquisition of desiccation tolerance in sycamore. Consistently, MsrB2 was reported to be involved in the regulation of redox status during the desiccation phase in *Acer* orthodox seeds, as its abundance was clearly correlated with changes in MetO and hydroxyl radical levels [17]. In developing *M. truncatula* seeds, distinct changes in transcript and protein levels revealed complex regulation of *MsrB* gene expression [35]. Interestingly, levels of both MsrB1 and MsrB2 were correlated with changes in the seed WC level (Supplementary Materials Figure S5). Considering that water stress does not affect MsrB1 and MsrB2 expression in young and mature Arabidopsis leaves [40], the changes in the abundance of MsrB1 and MsrB2 (Figure 3) were probably related to seed development-related events and not to drying down to as much as 50% WC during maturation. The fact that both the promoters of *MsrB1* and *MsrB2* include binding sites for transcription factors promoting seed development and morphogenesis support this hypothesis [34,56,57,58].

Developing seeds of Norway maple and sycamore are green [47] but are completely covered by an opaque brown seed coat. So far, plant MsrB1 and MsrB2 have been reported to be located in the stroma of leaf chloroplasts and protect this organelle against oxidative damage [40]. Given the widespread and non-tissue-specific localization of these proteins (Figure 4) in developing seeds, proplastids are possibly the target organelles of MsrB1 and MsrB2, but this needs further confirmation. MsrA was also found to accumulate in developing Norway maple seeds and was considered involved in dormancy induction [47]. Studies on sycamore seeds revealed that Met metabolism is associated with dormancy breaking [48]. Proteins linked to Met metabolism were significantly up-regulated during the germination of maize mutant seeds tolerant to drought [59]. Increased expression of the MsrA gene coincided with the reinduction of desiccation tolerance in germinated seeds of *M. truncatula* [60]. All together, these data are consistent with those of Kalemba and Stolarska’s report [34], emphasizing that Msrs, notably plastidial ones, are involved in the regulation of numerous plant developmental processes, including during seed formation.

### 4.3. Redox Pools in Orthodox and Recalcitrant Acer Seeds

The redox control of many physiological processes has been demonstrated in plants [22,23]. Previously, the ascorbate-glutathione cycle [11] and Prxs [12] have been characterized as important redox actors in the desiccation tolerance of developing *Acer* seeds. Here, we demonstrated that other elements of the redox network are involved in the determination of orthodox and recalcitrant phenotypes in two genetically related *Acer* species. A clear decrease in NAD level throughout maturation was reported uniquely in Norway maple (Figure 7), which is consistent with the progressive deceleration of metabolism observed during this phase in desiccation-tolerant seeds, and not in sensitive ones [5]. Sycamore seeds contained a lower NADP pool than did Norway maple seeds (Figure 8) and exhibited less reducing power in terms of available NADPH. The peak of NADPH-dependent reductase activity was reported in Norway maple seeds at the 14th–15th WAF (Figure 5). Considering that the maximal growth of Norway maple embryos occurs at the 14th WAF [47], the activity of NADPH-dependent reductases, seems essential in the control of seed development at this stage. The NADPH/NADP^+^ ratio is usually high, favoring the reduced form of NADP [61]. In Norway maple embryonic axes, the ratio peaked at 17th WAF (Figure 9). The signaling role of pyridine nucleotides [62] might be linked to desiccation tolerance acquisition, which was reported to begin in the 18th WAF [11]. Compared with recalcitrant seeds, orthodox Norway maple seeds underwent highly specific redox changes related to nucleotide pools (Figure 6 and Figure 8). The content and redox status of NADH/NAD^+^ and NADPH/NADP^+^ were shown to contribute to desiccation tolerance in dried *Acer* seeds [19]. This report indicates that the differences in NADH/NAD^+^ and NADPH/NADP^+^ redox couples are already being formed during maturation when desiccation tolerance is acquired by seeds.

The redox-related differences described in this report enabled us to point the features unique to orthodox Norway maple seeds, which include substantial changes in the MetO level, the presence of reduced and oxidized forms of MsrB2, and a dynamic AsA/DHA ratio, as well as high levels of glutathione, NADPH, and peaks in the activities of NADPH-dependent reductases. Importantly, the MetO level was positively correlated with changes in the activity of reductases, and there was a negative correlation between MetO and both protein levels and the AsA/DHA ratio (Figure S5), indicating that the Met to MetO switch is an element of the redox signaling network active during the Norway maple seed developmental program. These results indicate that the regulation of the Met redox switch in developing seeds is likely more dynamic, and probably more efficient in Norway maple than in sycamore.

### 4.4. Regeneration of Msrs

The Trx-dependent pathway is considered a major regeneration system for plastidial Msrs in plant tissues with active chloroplasts; however, Grx- and GSH/Grx-dependent mechanisms also act in MsrB1 regeneration [36,37]. Since MsrB1 is not reduced by Trxs [31,40], and based on the correlation matrix (Figure S5), it can be speculated that AsA and particularly GSH are involved in MsrB1 regeneration in sycamore seeds via intermediate redox actors. Importantly, AsA levels were the strongest factor correlated with the changes in MsrB2_red_ levels in Norway maple. AsA is able to reduce disulfide bridges, including protein mixed disulfides, and the reduction rate is concentration dependent [63]. Additionally, AsA is able to reduce sulfenic acid in proteins [64] and similarly to glutathione promote the reduced form of MsrB2 in Norway maple seeds [17]. However, further studies are required to determine the entire cascade of redox reactions in the regeneration of MsrBs in seeds. Levels of NADPH and NADP^+^ affected the expression of MsrB2_red_, whereas the levels of MsrB2_ox_ were seemingly driven by the NADH and NAD^+^ levels (Figure S5). Considering the Foyer-Halliwel-Asada cycle, NADH is required for AsA enzymatic regeneration, whereas NADPH enables GSH restoration [22]. Both pyridine nucleotides significantly affect the levels of MsrB2_red_, supporting our hypothesis concerning the involvement of AsA and GSH in MsrB2 reduction. In addition, MsrB2 was found in sycamore embryonic axes predominantly in the oxidized form (Figure 3B), and changes in the level of this form were correlated with AsA and glutathione contents, but not with the contents of NAD(P) forms (Figure S5). All reducing systems for the regeneration of Msrs eventually rely on NADPH availability [65]. In Norway maple, the activity of NADPH-dependent reductases was correlated with changes in all glutathione forms and was highly correlated with the DO of this compound (Figure S5), indicating that the GSH/GSSG switche further affects the reduction capacity in seed tissues. In sycamore cotyledons, NADH and NAD^+^ were positively correlated with components of the ascorbate-glutathione cycle, particularly AsA, and could thus very likely participate in the regeneration system of Msrs. In Norway maple, changes in NADH and NAD^+^ levels and in NADPH and NADP^+^ levels were correlated with all Asc forms, supporting that pyridine nucleotides modulate the efficiency of the ascorbate-glutathione cycle (Supplementary Materials Figure S5), which in turn affects the availability of essential reducing agents such as AsA and GSH. Importantly, the redox environment modulated by GSH DO further affected MsrB2_ox_ expression, indicating that GSH could be an important physiological reducing agent not only for MsrB1 [36], but also for MsrB2 in seeds of the two *Acer* species. Recently, Wojciechowska et al. [17] reported that the amount of reduced form of Norway maple MsrB2 increased when protein extracts were treated with AsA and GSH, supporting this hypothesis.

## 5. Conclusions

Controlled oxidation is critical for developmental transitions in plants, such as those occurring in seeds. In addition to cysteine redox switches, the reversible oxidation of Met to MetO is an important protein posttranslational modification involved in redox signaling [33]. Here, we show that the redox cellular environment strongly differs between developing Norway maple and sycamore seeds, which are categorized as orthodox and recalcitrant, respectively. In Norway maple, the high activity of NADPH-dependent reductases reported around the 14th WAF and the marked reducing conditions seem essential for proper development of orthodox seeds and acquisition of desiccation tolerance. Furthermore, we revealed more dynamic changes in the levels of MetO and MsrB2 in Norway maple than in sycamore, such as the much higher abundance of reduced MsrB2 in the former during the first stages of seed formation. The differences in MsrB2 abundance and redox pattern clearly differentiate Norway maple seeds from sycamore seeds, leading us to propose that this reductase is a new candidate for the establishment of desiccation tolerance. Among the various components involved in redox homeostasis, AsA by acting together with glutathione and reduced form of NAD(P), could provide the reducing power for the regeneration of plastidial MsrB activity in a non-photosynthetic context.

## Supplementary Materials

The following are available online at https://www.mdpi.com/2076-3921/9/12/1250/s1, Figure S1: Chromatogram with standards, Figure S2: Representative gels, Figure S3: Soluble proteins, Figure S4: Negative blots, Figure S5: Correlation matrixes.
