# Supplementary material for: Integration of MsrB1 and MsrB2 in the Redox Network during the Development of Orthodox and Recalcitrant Acer Seeds
Source: Antioxidants (Basel). 2020 Dec 9;9(12):1250. doi: 10.3390/antiox9121250 (PMC7763665; doi:10.3390/antiox9121250)
Supplement: Supplementary file 1 [file antioxidants-09-01250-s001.pdf]

Figure S1 Determinations of standsards of methionine sulfoxide (MetSO), methionine (Met), tyrosine (Tyr), and tryptophan (Trp) further used for the calibration curves. A representative chromatogram indicating peaks recognized at 214 nm is given.

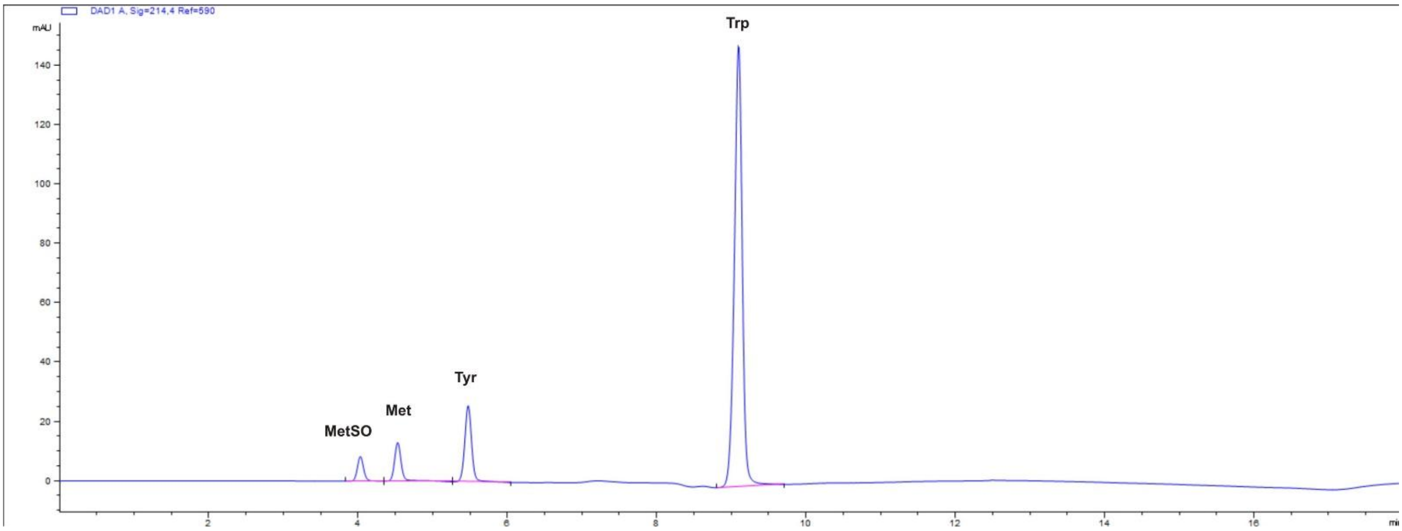

Figure S2 SDS-PAGE representative gels of protein extracts (20 μg) isolated form embryonic axes and cotyledons of Norway maple (A) and sycamore (B) seeds further used for Western blot analyses. Analyses concerned proteins originating from 11-23 weeks afte flowerinf (WAF). The Spectra^{TM} Multicolor Broad Range Protein Ladder marher (Thermo Scientific) was used to calculate the molecular weight. Gels were documented with (A) G:BOX Chemi XR5 instrument (Syngene, Cambridge, UK) and Coomassie Blue filter settings, (B) Fire Reader Gel Documentation System.

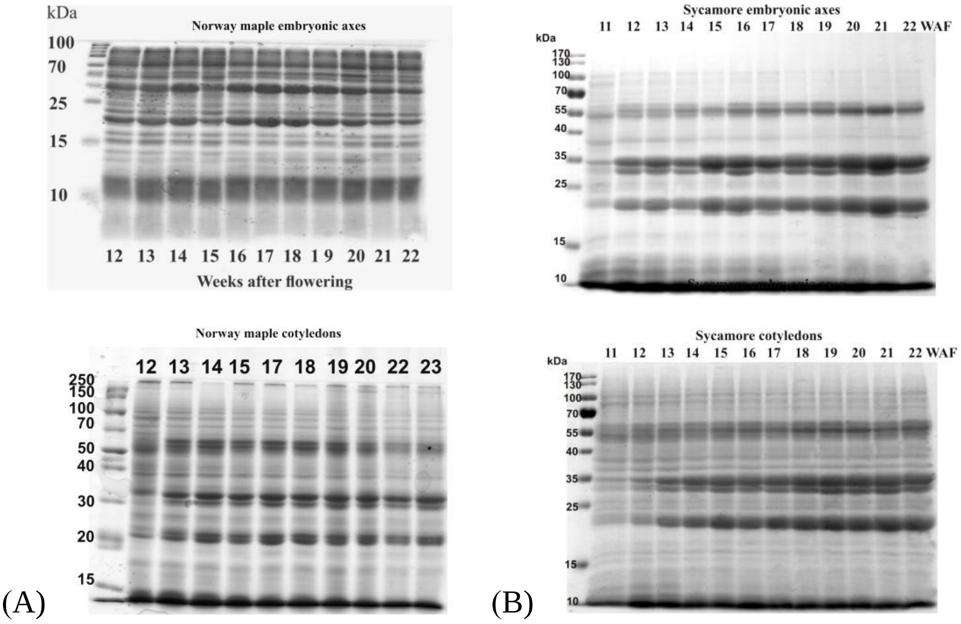

Figure S3 Changes in the levels of soluble protein in embryonic axes and cotyledons of *Acer platanoides* (squares) and *A. pseudoplatanus* (circles) developing seeds collected at the range of 11th - 23rd weeks after flowering. Data are the means of three independent replicates ± the standard error. Statistically significant differences are indicated with different letters (one-way ANOVA, followed by Tukey's test at $p < 0.05$). The capital letters refer to the *A. pseudoplatanus*.

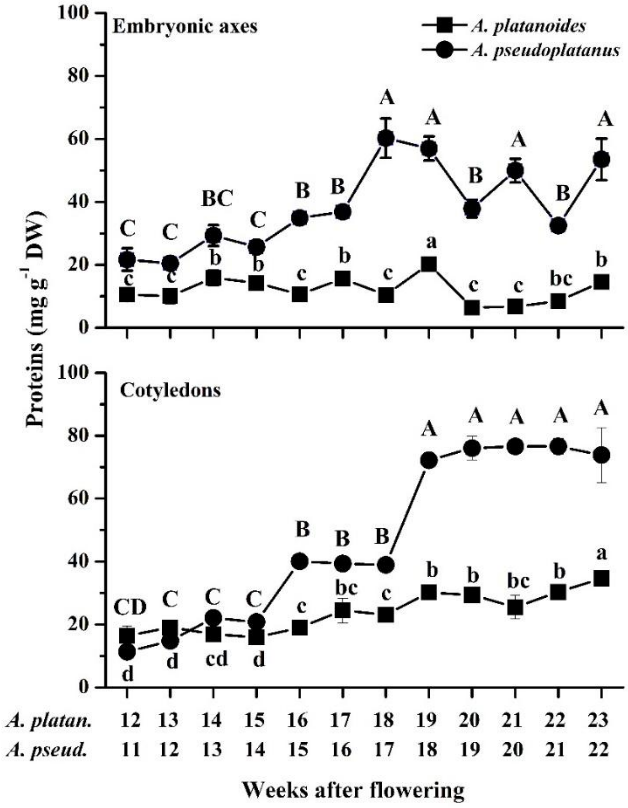

Figure S4 Western blots with negative results.

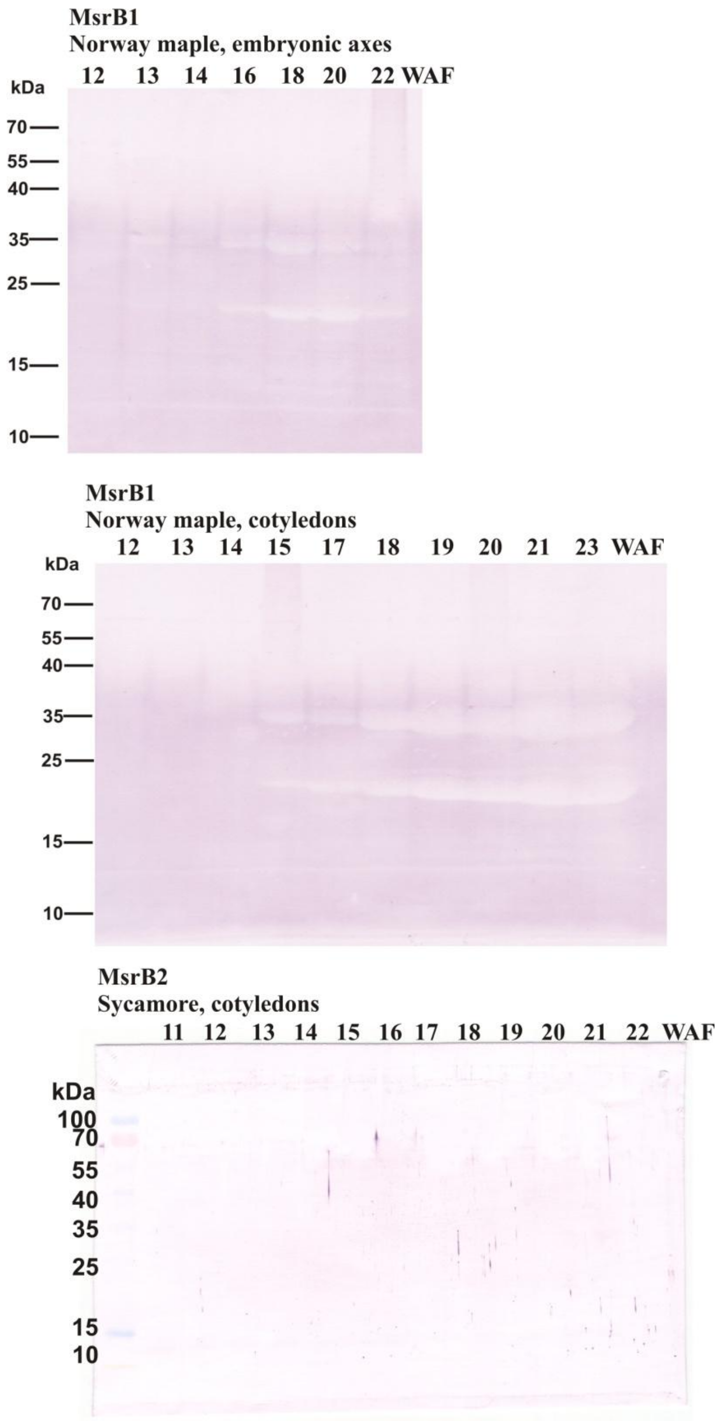

Figure S5 Correlation matrices calculated for embryonic axes (A) and cotyledons (B) of Norway maple (*Acer platanoides*), and embryonic axes (C) and cotyledons (D) of sycamore (*Acer pseudoplatanus*) based on levels of ascorbic acid (AsA), dehydroascorbic acid (DHA), total pool of ascorbate (Asc), Asa to DHA ratio (AsA/DHA ratio), levels of reduced glutathione (GSH), oxidized glutathione (GSSG), total pool of glutathione (TotalG), degree of oxidation of glutathione (DO), levels of reduced (NADH) and oxidized (NAD^+) nicotinamide adenine dinucleotide, NADH to NAD+ ratio ($NADH/NAD^+$ ratio), levels of reduced (NADPH) and oxidized ($NADP^+$) nicotinamide adenine dinucleotide phosphate, NADPH to NADP+ ratio ($NADPH/NADP^+$ ratio), water content (WC), levels of protein-bound methionine sulfoxide (MetO), the abundance of methionine sulfoxide reductase isoforms (MsrB1, MsrB2), activity of NADPH-dependent reductases (TotR). Crossed numbers indicate non-significant correlation (P > 0.05).

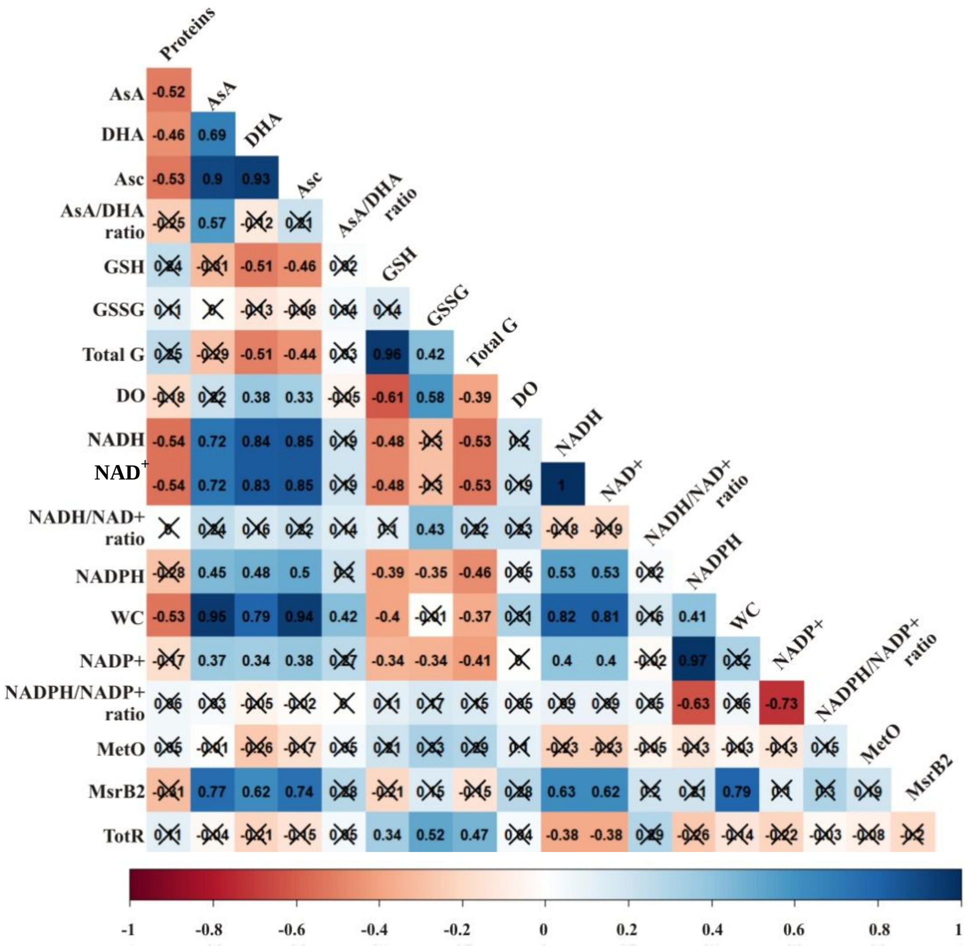
Proteins
AsA
DHA
Asc
AsA/DHA ratio
GSH
GSSG
Total G
DO
NADH
NAD+
NADH/NAD+ ratio
NADPH
WC
NADP+
NADPH/NADP+ ratio
MetO
MsrB2
TotR
-1 -0.8 -0.6 -0.4 -0.2 0 0.2 0.4 0.6 0.8 1

(A)

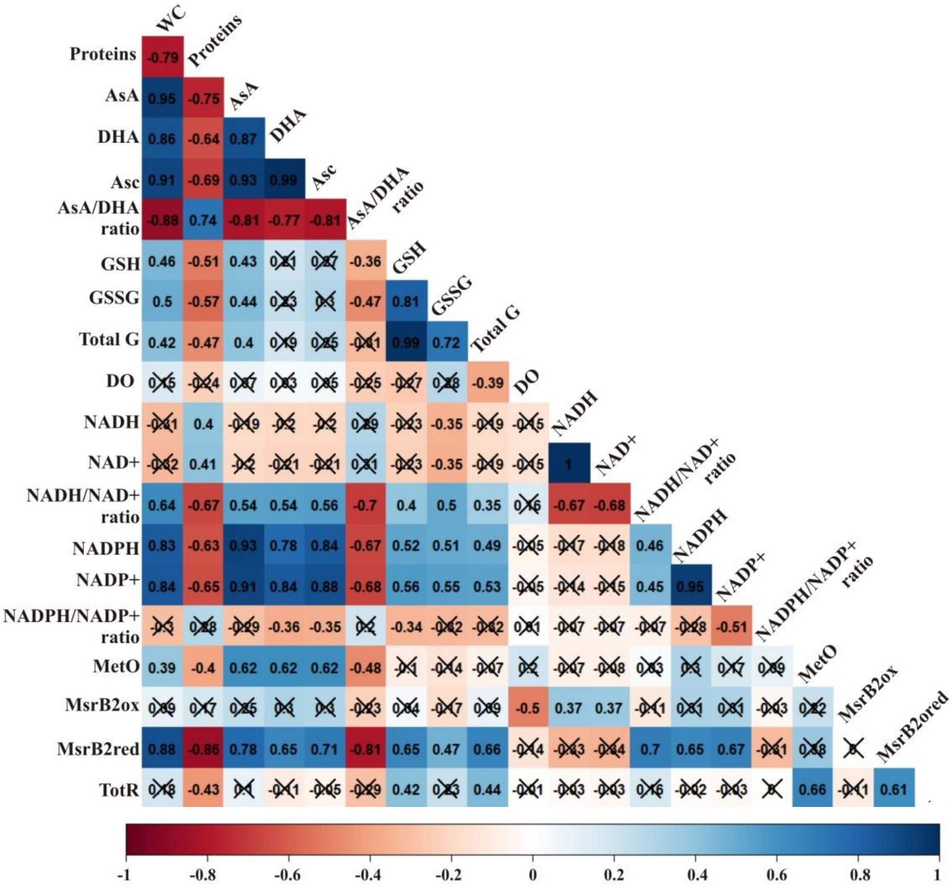
WC
Proteins
AsA
DHA
Asc
AsA/DHA ratio
GSH
GSSG
Total G
DO
NADH
NAD+
NADH/NAD+ ratio
NADPH
NADP+
NADPH/NADP+ ratio
MetO
MsrB2ox
MsrB2red
TotR
-1 -0.8 -0.6 -0.4 -0.2 0 0.2 0.4 0.6 0.8 1

(B)

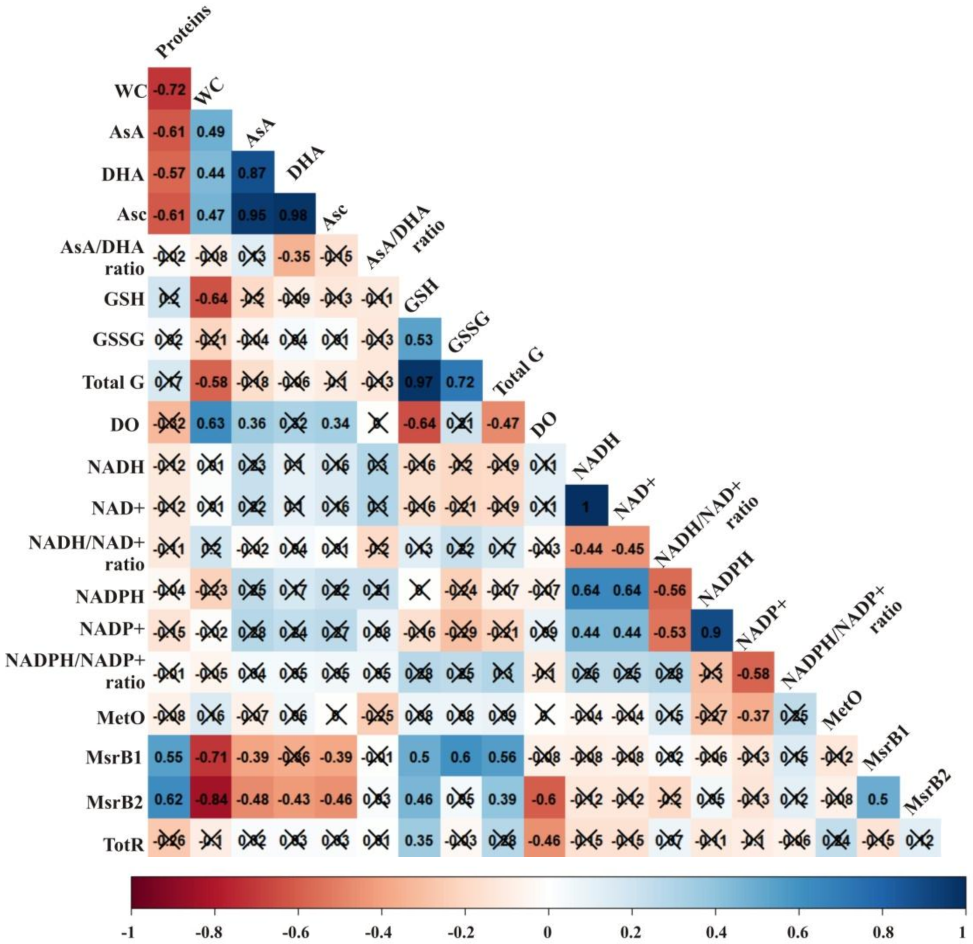
Proteins
WC
AsA
DHA
Asc
AsA/DHA ratio
GSH
GSSG
Total G
DO
NADH
NAD+
NADH/NAD+ ratio
NADPH
NADP+
NADPH/NADP+ ratio
MetO
MsrB1
MsrB2
TotR
-1
-0.8
-0.6
-0.4
-0.2
0
0.2
0.4
0.6
0.8
1

(C)

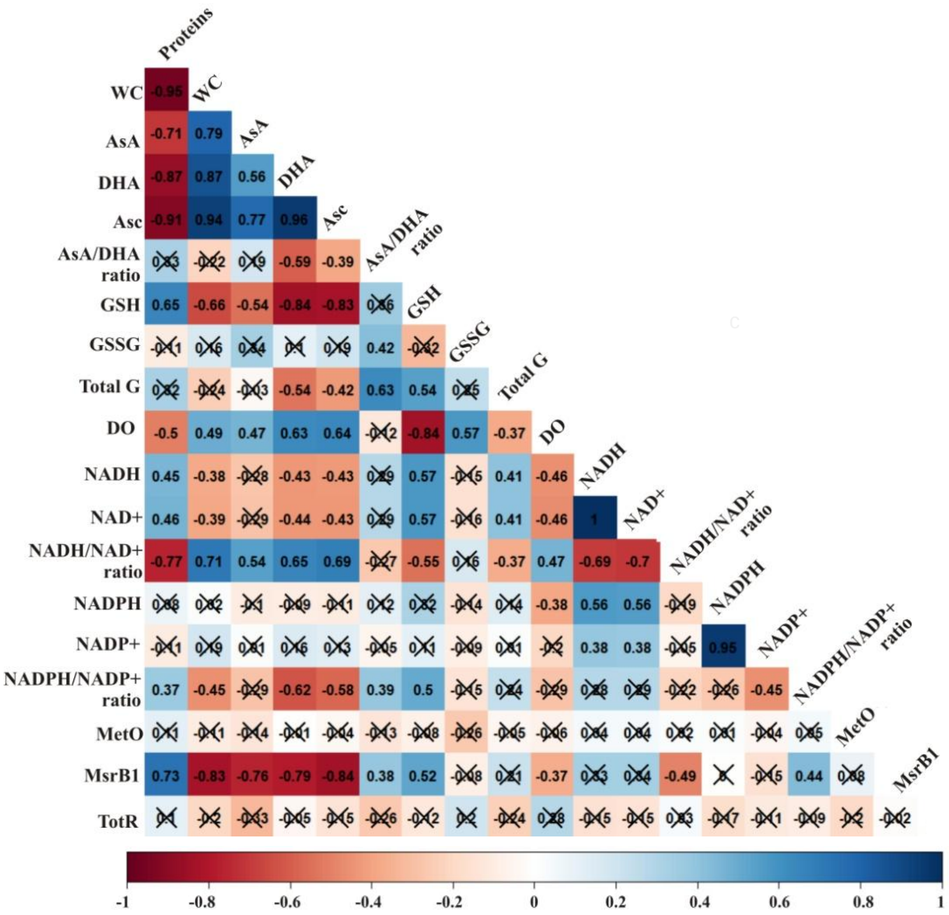
Proteins
WC
AsA
DHA
Asc
AsA/DHA ratio
GSH
GSSG
Total G
DO
NADH
NAD+
NADH/NAD+ ratio
NADPH
NADP+
NADPH/NADP+ ratio
MetO
MsrB1
TotR
-1
-0.8
-0.6
-0.4
-0.2
0
0.2
0.4
0.6
0.8
1

(D)
